# Implicações do governo Bolsonaro à prevenção e ao tratamento do HIV/aids

**DOI:** 10.1590/0102-311XPT188723

**Published:** 2024-11-22

**Authors:** Tiago Sousa Paiva, Daniel Canavese de Oliveira, Adriano Maia dos Santos, Deise Lisboa Riquinho

**Affiliations:** 1 Universidade Federal do Rio Grande do Sul, Porto Alegre, Brasil.; 2 Instituto Multidisciplinar em Saúde, Universidade Federal da Bahia, Salvador, Brasil.

**Keywords:** HIV, Prevenção de Doenças, Terapêutica, Política de Saúde, HIV, Disease Prevention, Therapeutics, Health Policy, VIH, Prevención de Enfermedades, Terapéutica, Política de Salud

## Abstract

Neste ensaio, nosso objetivo é debater acerca das implicações do projeto político da extrema-direita no Brasil, na figura de Jair Bolsonaro, no que tange aos retrocessos provocados na política e nas ações para a prevenção e tratamento do HIV/aids no país. São objetos da nossa análise o desmantelamento da política de aids; o conservadorismo de cunho religioso; a retirada de direitos das pessoas que vivem com HIV; e a produção das *fake news*, no contexto da epidemia do HIV/aids. Foi possível observar implicações na política e nas ações de prevenção e tratamento, por exemplo a ausência de propostas para enfrentar problemas estruturais que atingem grupos vulnerabilizados, como a pobreza, o estigma e as violações aos direitos fundamentais.

## Abstract (en)

Our objective in this essay is to discuss the implications of the political project of the far-right in Brazil, in the figure of Jair Bolsonaro, with regard to the setbacks caused in the policy and actions for the prevention and treatment of HIV/AIDS in Brazil. The objects of our analysis are the dismantling of the AIDS policy; religious-based conservatism; the withdrawal of rights from people living with HIV and the production of fake news, in the context of the HIV/AIDS epidemic. It was possible to observe implications in the policy and in the actions of prevention and treatment, such as the absence of proposals that sought to address structural problems that affect vulnerable groups, including poverty, stigma and violations of fundamental rights.

## Abstract (es)

Este ensayo pretende discutir las implicaciones de la política de extrema derecha en Brasil bajo la figura de Jair Bolsonaro sobre las acciones para la prevención y el tratamiento del VIH/sida en el país. Los objetos de este análisis son el desmantelamiento de la política del sida; el conservadurismo de carácter religioso; la retirada de derechos de las personas que viven con el VIH; y la producción de noticias falsas en el contexto de la epidemia del VIH/sida. Hubo implicaciones en las acciones de la política y en la prevención y tratamiento, como la ausencia de propuestas que buscaron abordar problemáticas estructurales que afectan a los grupos vulnerables, como la pobreza, el estigma y las violaciones de los derechos fundamentales.

## Introdução

Alicerçado na perspectiva interpretativista, este ensaio tem como objetivo compreender os fenômenos decorrentes da ascensão do projeto político da extrema-direita brasileira e suas implicações nas ações de prevenção do HIV/aids, no Brasil. Tal perspectiva propõe entender os fenômenos sociais a partir da interação entre as pessoas e do contexto histórico, social e cultural. Diante disso, cada elemento da sociedade é parte de uma totalidade que nos constitui, sendo a realidade em que estamos inseridos o resultado da soma de fatores políticos, econômicos e de movimentos de transformação da sociedade ocorridos ao longo do tempo ^1^.

Este artigo tem como ponto de partida os fatos políticos que permearam os governos de Dilma Rousseff e Michel Temer ^2^, compreendidos como relevantes para o contexto que pretende-se analisar. Em 2011, após a eleição da primeira mulher presidenta do Brasil, o país ainda sofria impactos da desaceleração econômica produzida pela crise mundial do capitalismo, em 2008 ^3^. Esse contexto tornou o cenário para a governabilidade de Dilma ainda mais desafiador e foi percebido o recrudescimento, no âmbito legislativo, de grupos denominados como bancada fundamentalista religiosa e bancada da bala, sendo este último, hegemonicamente, composto por agentes da segurança pública e por simpatizantes de políticas armamentistas ^4^. Em diversas ocasiões, tais grupos fomentaram propostas que visavam impedir que ações de prevenção e tratamento do HIV, especialmente quando direcionadas à comunidade LGBTQIA+, fossem desenvolvidas no âmbito do executivo e das políticas públicas, mesmo que naquele grupo estivessem as pessoas mais vulnerabilizadas e estigmatizadas pela epidemia ^5^.

Um exemplo concreto foi a censura imposta, após críticas e pressões do Congresso Nacional, à campanha para a prevenção do HIV, durante o carnaval de 2012. Além disso, no ano seguinte, outra ação midiática voltada à prevenção do HIV, tendo como foco as trabalhadoras do sexo - com o slogan *Sou Feliz Sendo Prostituta* -, foi interrompida prematuramente após duras críticas de grupos radicais. O objetivo da campanha, todavia, era ampliar o protagonismo desse grupo historicamente estigmatizado no enfrentamento da epidemia do HIV/aids ^6^.

Esses episódios são precisas violações ao direito à prevenção ^7^, pois impossibilitam o acesso a estratégias que produzem respostas às vulnerabilidades ^8^. Ademais, é sabido que, quando são engendradas interdições ao debate sobre sexualidade ou as diferentes maneiras das expressões de vida, são reforçados os estereótipos, os estigmas e os preconceitos, considerados barreiras para a superação do HIV/aids ^9^.

Apesar da postura conservadora do legislativo nacional, o governo Dilma construiu alianças, por meio da coalizão político-partidária, com partidos do chamado Centrão, representados pela extrema e centro-direita ^10^. Com tensões de ordem política e econômica, o segundo mandato da presidenta foi marcado pelo aumento de medidas de austeridade fiscal e pela abertura do setor de saúde ao capital estrangeiro, sendo a última medida muito criticada por pesquisadores, profissionais da saúde e defensores do Sistema Único de Saúde (SUS) ^11^. Após as sucessivas denúncias de corrupção, foi favorecida ainda mais a reorganização de forças ultraconservadoras e de extrema-direita, no país ^12^. Diante disso, Dilma Rousseff foi deposta do cargo de presidenta com um golpe parlamentar, em 2016 ^13^ ^,^ ^14^. Nesse cenário, emergiu uma agenda para a política de HIV/aids, na qual não foram observadas propostas que visavam ao cuidado integral à saúde de pessoas impactadas pela epidemia ^15^.

Com a saída da presidenta, quem assumiu a Presidência da República foi o seu vice, Michel Temer, político de um partido que tem se equilibrado no poder desde o início do processo de redemocratização do Brasil, atuando na perspectiva da política de coalização, tanto em governos de direita quanto de esquerda ^16^. Outrossim, cientistas políticos defendem que Michel Temer conspirou para a queda de Dilma e que ele e seu partido desenhavam um programa paralelo de governo ^17^.

“Uma Ponte Para o Futuro” foi nome dado para o programa de governo após o *impeachment*, com um aceno explícito para o mercado financeiro e sinalizando para a elite econômica brasileira que Temer seria a pessoa que cumpriria com a agenda das contrarreformas, indispensável para o capitalismo e com impacto negativo nas ações de prevenção do HIV/aids. Nesse contexto, foram percebidas sucessivas violações de direitos, sobretudo do direito à saúde ^16^.

Nesse esteio, cabe mencionar a aprovação da *Emenda Constitucional 95/2016*, que criou um teto para investimentos públicos, impactando diretamente no setor de saúde ^18^. Desde então, a política de HIV/aids no Brasil passou a sofrer sucessivas descontinuidades de ações importantes para o enfrentamento de problemas estruturais que, mesmo na atualidade, são obstáculos para a superação da epidemia ^15^. Ainda, diante do contexto de retrocessos na política, passadas duas semanas da posse de Michel Temer, a delegação brasileira anunciou que não participaria da conferência internacional, organizada anualmente pela Organização das Nações Unidas (ONU), em que se discutiriam estratégias para o enfrentamento da epidemia. Decorridos dois anos do mandato e com a chegada das eleições de 2018, abriu-se o caminho para a ascensão de um projeto político e social que agregou autoritarismo e neoliberalismo, com destaque para a agenda conservadora e reacionária ^19^. Foi em meio ao medo e a ameaças de retiradas de direitos à saúde que a política de HIV/aids entrou na era Jair Bolsonaro ^20^.

## Depois da “ponte para o futuro”

O fenômeno político da extrema-direita brasileira compõe um movimento internacional ^21^ denominado de onda conservadora, o qual encontrou em Bolsonaro um presidenciável com a retórica de tal regime ^22^. Na atualidade, grupos políticos ligados à extrema-direita internacional estão ganhando espaço, por exemplo, no Parlamento Europeu ^23^, haja vista os resultados da última eleição, e, nos Estados Unidos, que revive a possibilidade da vitória de Donald Trump, nas eleições presidenciais de 2024 ^24^.

No cenário nacional, a extrema-direita brasileira organiza-se como atuante digitalmente, com um forte tom populista, e tem se apropriado do aparato do Estado para pôr em prática seu projeto de poder ^25^. A usurpação do Estado não precisa ocorrer, necessariamente, por meio de força ou de um golpe clássico, ela pode ser observada quando líderes de tendência autoritária assumem o poder e utilizam-se do aparato estatal para corroer a democracia ^25^. Essas características foram marcantes durante a gestão de Jair Bolsonaro ^26^ ^,^ ^27^, com destaque para a utilização do aparato militar. A partir do processo democrático eleitoral de 2018, foi possível acompanhar um número significativo de militares ocupando o centro do poder político ^28^.

O governo Bolsonaro superou o período da Ditadura Militar em número de militares ocupando o primeiro escalão, segundo dados identificados pelo Tribunal de Contas da União ^29^, caracterizando-se não como um governo militar, mas como um governo que deu maior destaque para os militares ^30^ ^,^ ^31^. Ao relembrar os símbolos escolhidos pelo governo Bolsonaro, a exemplo da militarização do aparato do Estado, são suscitadas memórias de um período da história no qual discursos fundamentalistas dificultavam o avanço da prevenção ao HIV/aids ^30^. No período da ditadura, a escola era entendida como um espaço destinado à consolidação da moral e dos “bons costumes”, desconsiderando as singularidades dos estudantes e a necessidade do desenvolvimento da cidadania. Temas que permeavam a sexualidade eram considerados tabus e passíveis de censura. Remonta-se a esse período o projeto das escolas cívico-militares, projeto interministerial que envolveu o Ministério da Justiça e o Ministério da Educação, sendo observada uma forte interferência dos militares na educação ^32^.

Uma análise a partir de um dos critérios utilizados pelo projeto em questão, como a obrigatoriedade de mudanças na aparência física, buscando uma homogeneidade em cortes de cabelo, ou penteados, e vestimentas, permite-nos inferir que um dos princípios básicos da nossa *Constituição Federal* estava sendo violado, o princípio do direito à Educação, limitando o acesso de diferentes perfis de jovens e de adultos à escola a partir de uma clara instrumentalização do Estado. Ademais, nem todos os estudantes se adequam às rígidas normas que são estabelecidas pelas forças armadas, sendo cabível, portanto, tecermos a consideração de que o projeto em questão é operacionalizado em espaços públicos (escolas). Para esses estudantes, quais caminhos ou acessos à Educação seriam oportunizados? Esse mesmo tipo de análise também é feito por pesquisadores do campo da Educação ^27^. Outra discussão abordada por nós é que o exercício pleno da cidadania, produzido, também, pela convivência escolar, pode enfrentar barreiras, principalmente para aqueles estudantes que não se enquadram nesses padrões e, por isso, ficam limitados de acessar à escola (espaço público). Diante disso, como seria o acesso à escola cívico-militar de jovens travestis, transexuais ou que não performam dentro da cisheteronormatividade? Consideramos que o cotidiano escolar deve ser universal e um direito para todas as pessoas, colaborando para a superação de situações desafiadoras existentes na sociedade ^33^ ^,^ ^34^, a exemplo das situações que produzem suscetibilidade ao HIV/aids. Com efeito, a Reforma do Ensino Médio, idealizada ainda no governo de Michel Temer e implementada no governo de Jair Bolsonaro, é compreendida por especialistas como uma contrarreforma neoliberal capaz de capturar a vida dos jovens e produzir ausência de consciência acerca de temas que impactam a coletividade, por exemplo o HIV/aids ^35^. Somando-se ao projeto de militarização do ensino público e a Reforma do Ensino Médio, percebeu-se o crescimento de censuras nos debates sobre sexualidade e gênero, dentro das escolas durante a gestão Bolsonaro ^36^.

Sobre isso, frisamos o projeto de lei “Escola sem Partido” ^37^. Com o argumento de que a Proposta de Emenda à Constituição (PEC) pretende proteger as crianças e os jovens da “Ideologia de Gênero” ^38^, o projeto tenta silenciar e punir educadores que abordam temas diferentes aos de uma sociedade ultraconservadora ^9^. Esse tipo de iniciativa, caso aprovada, dificulta a produção de perspectivas críticas acerca do uso de preservativos e de outras ferramentas utilizadas para prevenção durante as relações e envolvimentos sexuais e, consequentemente, serão ampliados os contextos de vulnerabilização ^39^.

De fato, a ofensiva contra a diversidade sexual teve início antes mesmo de Bolsonaro ser eleito, e notícias falsas, como o chamado “Kit Gay”, eram espalhadas ^40^. No entanto, ainda no período eleitoral, um dos ministros do Tribunal Superior Eleitoral proferiu a decisão sobre a suspensão de links de sites e redes sociais com a expressão “Kit Gay”. Narrativas como essas estimulam a produção de afetos que criam inimigos no imaginário de grupos extremistas e reacionários, fato que poderá fomentar a aversão à diversidade e violências ^41^. Em uma pesquisa realizada com o objetivo de investigar os discursos de ódio nas *fan-pages* à época, o então deputado federal, Jair Messias Bolsonaro, foi observado pela incivilidade e pelos discursos discriminatórios sobre diferentes temas, dentre eles, orientação sexual e identidade de gênero, impulsionando violações aos direitos fundamentais das pessoas, além de representar ameaça à democracia e à vida ^42^.

Além dos sucessivos ataques e violações de direitos no campo da Educação, após vencer as eleições presidenciais, o Governo Bolsonaro implementou no país uma agenda econômica ultraneoliberal ^43^, com repercussões negativas na política de seguridade social. Em verdade, as políticas de seguridade social são essenciais, principalmente, para o enfrentamento do estigma que pessoas que vivem com HIV sofrem no mundo do trabalho ^44^. Entretanto, em seis meses de governo, em julho de 2019, a gestão Bolsonaro vetou o *Projeto de Lei* (PL) *nº 10.159/2018* ^45^ que visava à dispensa de reavaliação pericial de pessoas vivendo com HIV que foram aposentadas por invalidez.

Para a Associação Brasileira Interdisciplinar de aids (ABIA), em nota publicada que analisou os 100 dias do governo, ao realizar o veto, não foram levados em consideração o estigma e o preconceito que pessoas que vivem com HIV sofrem nos espaços de atividade profissional ^46^. No mesmo documento publicado pela ABIA, outros retrocessos na política de aids foram denunciados, como o desmantelamento do departamento responsável por promover políticas de enfrentamento à aids no Ministério da Saúde. Com o *Decreto nº 9.795* ^47^, o Departamento de HIV/aids, Tuberculose, Hepatites Virais e Infecções Sexualmente Transmissíveis foi inserido em um setor mais amplo, o qual objetiva promover ações voltadas para outras enfermidades, como a tuberculose e a hanseníase. Diante disso, alguns especialistas alertaram para o risco de perda do reconhecimento e da qualidade das ações, tendo em vista a atenção necessária para as especificidades de cada uma dessas doenças ^46^.

Além disso, frente às mudanças na estrutura do Ministério da Saúde, foi observada a ausência de diálogo com os movimentos sociais, historicamente protagonistas e fundamentais no enfrentamento da epidemia de HIV/aids ^48^. Tais alterações impostas à política de prevenção ao HIV e a ruptura do governo com movimentos sociais produziram inequivocamente respostas ineficientes, dada a magnitude do cenário epidemiológico.

Posto isso, argumentamos que, com a eleição de Jair Bolsonaro, a participação popular na política de prevenção ao HIV sofreu um profundo retrocesso, seja pela ausência de iniciativas para o enfrentamento dos problemas que atingem as pessoas mais impactadas pela epidemia ou pela falta de participação em conselhos e coordenadorias que se ocupam do planejamento dos rumos dessa política estratégica no SUS.

Nesse panorama estarrecedor, o contexto de contrarreformas só não foi mais grave, porque os(as) servidores(as) públicos(as), logo nos primeiros meses do governo Bolsonaro, agiram para impedir os avanços do autoritarismo e retrocessos que estavam em curso. Mesmo o governo utilizando instrumentos formais de repressão e assédio, como implementação de processos administrativos e disciplinares (PAD) e exonerações de cargos, os(as) servidores(as) valeram-se de estratégias individuais e coletivas para se contraporem às contrarreformas. Um exemplo disso foi apresentado em um estudo que entrevistou 165 servidores(as) públicos(as) federais durante a gestão do governo Bolsonaro. Na ocasião, os(as) agentes públicos(as) mencionaram estratégias de resistência, por meio de registros e discordância formal das iniciativas do governo; denúncias aos veículos de imprensa; e movimentos de saída dos órgãos de origem para departamentos considerados mais “seguros” ^49^. Além disso, a mídia e os movimentos sociais tiveram um papel fundamental na denúncia dos retrocessos impostos pela gestão Bolsonaro, no campo das políticas públicas de saúde ^50^.

Frente aos sucessivos retrocessos, nem mesmo o componente biomédico deixou de sofrer impactos. No final de 2020, o governo de Jair Bolsonaro permitiu expirar o contrato com a empresa responsável pela realização de exames de HIV e hepatite C. Consequentemente, as novas solicitações de exames foram suspensas, impactando na qualidade de vida e no acesso ao tratamento de mais de 900 mil pessoas que vivem com HIV e/ou hepatite C, no Brasil e que necessitam dos exames para o monitoramento da carga viral. A informação do vencimento do contrato foi publicada em nota informativa divulgada no dia 2 de dezembro, pelo Departamento de Doenças de Condições Crônicas e Infecções Sexualmente Transmissíveis ^5^, um dia após o *Dia Mundial de Luta Contra a Aids*. Nesse mesmo ano, um documento produzido pelo Programa Conjunto das Nações Unidas sobre HIV/aids (UNAIDS) denunciou a interrupção dos serviços de tratamento e prevenção da infecção no Brasil. Nessa conjuntura, pesquisadores alertaram para o cancelamento de consultas e para a dificuldade no acesso à PrEP (profilaxia pré-exposição) durante a pandemia da COVID-19 ^51^.

Em outro vislumbre, é impossível produzir reflexões acerca dos cenários que permearam a política de prevenção ao HIV no Brasil durante a gestão de Bolsonaro sem contextualizar o período da pandemia da COVID-19. Emoldurados por um cenário econômico de austeridade fiscal e somados a políticas de um governo fundamentalista religioso e de extrema-direita, investimentos públicos foram reduzidos, dificultando a universalidade e a integralidade no cuidado da saúde. A esse cenário, une-se a grave estratégia negacionista ^52^ utilizada pelo governo durante o gerenciamento da pandemia que deu espaço para associações entre a vacina da COVID-19 e o HIV ^53^.

No dia 21 de outubro de 2021, o presidente, em *live* semanal realizada pelo Facebook, compartilhou informações falsas que relacionavam a vacina contra a COVID-19 à aids. Na ocasião, Bolsonaro, sem citar ou apresentar a fonte, lê a seguinte notícia: “*Relatórios oficiais do governo do Reino Unido sugerem que os totalmente vacinados estão desenvolvendo síndrome da imunodeficiência adquirida muito mais rápido do que o previsto*” ^54^. Em resposta, entidades como a Sociedade Brasileira de Infectologia e a UNAIDS se manifestaram, por meio de nota, repudiando o relato, o qual não tinha qualquer embasamento científico. Por conseguinte, consideramos que esse tipo de vinculação recrudesce estigma e discriminação, ambos historicamente ancorados nas pessoas que vivem com HIV, e instigam no imaginário social o medo de uma doença que, no passado, foi uma sentença de morte ^55^.

O cenário de proliferação de desinformações acerca da vacina da COVID-19 tornava-se ainda pior com o aumento do número de óbitos em decorrência das complicações da aids em pessoas que tiveram o diagnóstico tardio durante a pandemia ^56^. Para pesquisadores do campo da Saúde Coletiva, a má condução da pandemia da COVID-19 e o reflexo em outras enfermidades, a exemplo do HIV/aids, também estiveram relacionados ao despreparo técnico e às medidas negacionistas que emergiram dos gestores da saúde no âmbito federal, com aval do governo Bolsonaro ^57^. Ainda sobre o contexto pandêmico, foi possível observar debates acerca da proliferação de desinformações ^58^, as chamadas *fake news*, percebidas nos processos de comunicação entre determinados grupos que usam, principalmente, os recursos digitais para a disseminação de mentiras. Tal fenômeno é característico da era da pós-verdade e foi percebido, no contexto da pandemia, como uma estratégia da extrema-direita para negar a ciência ^59^. O termo pós-verdade tem sua origem no dramaturgo e romancista estadunidense Steve Tesicho que, em 1992, escreveu o livro *O Governo de Mentiras* ^60^, a fim de problematizar o contexto social e político dos Estados Unidos, naquele momento histórico. Em 2016, o *Dicionário Oxford* escolheu a pós-verdade como a palavra do ano e descreveu que o uso do termo “*se relaciona ou denota circunstâncias nas quais fatos objetivos têm menos influência em moldar a opinião pública do que apelos à emoção e a crenças pessoais*” ^61^. Esse tipo de fenômeno é capaz de produzir indivíduos assujeitados, mortos socialmente e sem condições de serem revolucionários, pois, a verdade é vista como mais um delírio e o valor epistemológico da ciência é negado ^62^. Com isso, é possível inferir que o governo Bolsonaro utilizou a mentira como estratégia política para desacreditar os agravos da COVID-19, fato percebido quando são apresentados argumentos reacionários para impedir a visibilidade de indivíduos dissidentes ou que performam fora dos padrões conservadores ^63^.

Em outro viés, as mudanças realizadas pelo mesmo governo no programa de transferência de renda Bolsa Família precisam ser compreendidas à luz do impacto das políticas sociais na superação dos cenários produtores de vulnerabilização ao HIV/aids. A gestão do ex-presidente Jair Bolsonaro propôs mudanças, em 2021, e o Bolsa Família foi substituído pelo Programa Auxílio Brasil, por meio do qual, além de alteradas as regras para o repasse monetário às famílias, foram modificados alguns condicionantes para a manutenção, dentre eles, a comprovação da vacinação de crianças de zero a seis anos e a adesão às consultas de pré-natal, dificultando ainda mais a prevenção do HIV. Nesse cenário, foi possível notar um espaço significativo de reforço aos grupos de ativistas anti-vacinas ^64^, fato que colaborou para retrocessos ao Programa Nacional de Imunização (PNI), percebidos mesmo após a derrota da extrema direita, nas eleições presidenciais de outubro de 2022.

Sabemos que o negacionismo à ciência, somado ao contexto da pós-verdade e à ideia de uma sociedade ultraliberal, colocou em risco a vida das pessoas, durante a gestão de Bolsonaro. Antes das mudanças propostas pelo referido governo entrarem em vigor, um estudo desenvolvido por duas pesquisadoras brasileiras comparou famílias beneficiadas e não beneficiadas do Bolsa Família e evidenciou uma redução de 16% da mortalidade de crianças entre 1 e 4 anos nas famílias que receberam o repasse entre 2006 e 2015. A diminuição se mostrou ainda mais significativa em famílias em que a transferência de renda foi realizada para mães autodeclaradas negras e que viviam em comunidades com o processo de pauperização acentuado. Em relação às mulheres negras, o *Boletim Epidemiológico* de 2023 demonstrou um aumento do número de mortes nesse grupo ^65^. Diante desse cenário epidemiológico, o HIV tem se desenvolvido e, com isso, podemos perceber que, na medida em que ocorrem retrocessos nas políticas públicas voltadas para o enfrentamento de problemas estruturais como a extrema pobreza, grupos específicos populacionais passam a ter menor acesso às estratégias de prevenção ao HIV e de cuidado, ampliando os casos de óbitos relacionados à aids.

O desmonte das ações de prevenção e tratamento do HIV/aids durante o governo Bolsonaro, com o mesmo passo da corrosão da democracia, afetou gravemente o SUS. Reiteramos, pois, o desmantelamento e as ameaças dirigidos ao SUS, os quais também produzem intimidações à histórica luta para conter a epidemia no Brasil ^66^.

Findo o pleito eleitoral de 2022, a gestão Bolsonaro teve o seu fim, com a organização de uma frente ampla e política articulada para interromper as violações em curso, simbolizando o restabelecimento da ordem democrática e do direito à vida. Diante disso, emergem questionamentos como: mesmo após o advento de um governo eleito democraticamente, o qual carrega consigo a bandeira das múltiplas lutas sociais, como refrear as ameaças ao SUS e à democracia, que tanto afetam as discussões sobre o HIV/aids? Mais do que nunca, deve-se investir no fortalecimento das instituições de Estado e na democracia. Além disso, a resposta pode ser encontrada, em parte, no processo de compressão construído durante a tessitura deste ensaio ^67^ ^,^ ^68^.

Destarte, uma breve guinada ao passado faz-se mister, nas manifestações de 2013, quando a extrema-direita se organizou, tanto nas ruas quanto digitalmente, encontrando, posteriormente, na figura política de Bolsonaro um catalisador de suas ideias ultraconservadoras e reacionárias. Nesse contexto, a ideologia e o movimento conservador que protagonizou a cena política no século 21 transcende a própria figura de Jair Bolsonaro ^69^. Com isso, a extrema-direita independe desse indivíduo - o Bolsonaro - para que ataques às conquistas historicamente obtidas ao longo das quatro décadas da política de enfrentamento da epidemia de HIV/aids sejam perpetrados.

A agenda desenhada desde 2013 e com maior intensidade de 2018 em diante, com a ascensão ao poder de governos de extrema-direita, produziu inúmeros retrocessos e ameaças no Brasil. Portanto, mesmo que, ainda, os recursos biomédicos estejam sendo disponibilizados para a prevenção do HIV/aids, como a PrEP e PEP (profilaxia pós-exposição), pessoas que vivem em cenários de violação de direitos são as mais impactadas por essa epidemia ^70^. É importante frisar que os impactos dos projetos políticos e sociais da extrema-direita afetaram o cenário epidemiológico do HIV/aids. A ausência ou debilidade de políticas públicas ou a organização propositada de ações de desinformação são graves; elas incitam ainda mais o movimento do estigma e da discriminação os quais, sabidamente, são um dos maiores desafios para a superação da epidemia e das situações de morte. Como todo processo de determinação de saúde, infelizmente, a carga da iniquidade demonstra que padecem ainda mais as pessoas com os marcadores na perspectiva da interseccionalidade, seja a questão da raça/cor e etnia, da origem geográfica, da renda, da religiosidade, da deficiência, da identidade de gênero e da orientação sexual.

## Considerações finais

No cenário internacional, o projeto político da extrema-direita continua ganhando força e, no Brasil, deu espaço para a figura de Bolsonaro, fazendo emergir tensões e retrocessos no campo político e social, de modo que produziu vulnerabilidades a grupos que historicamente são impactados pela epidemia do HIV/aids. Esse fato refletiu-se na ausência de propostas que buscassem enfrentar os problemas estruturais que permeiam o cenário epidemiológico do HIV/aids, como a LGBTfobia, a pobreza e o estigma. Portanto, tal contexto aponta para a necessidade de uma constante vigilância e reafirmação do compromisso de uma frente ampla que defenda a democracia, a vida e o SUS. Com isso, as instituições democráticas fortalecidas continuarão barrando o avanço de projetos como os do governo Bolsonaro e de qualquer outro grupo político que vislumbre possibilidades para a implementação da agenda da extrema-direita no Brasil.
